# Unique and cumulative effects of different subtypes of sleep problems on burnout among Chinese nurses

**DOI:** 10.3389/fpsyg.2024.1483771

**Published:** 2024-10-23

**Authors:** Ping-Zhen Lin, Yong-Sen Lin, Xu Wang, Lan-Lan Chen, Yan-Yan Lin, Bi-Yu Wu

**Affiliations:** ^1^Nursing Department, Quanzhou First Hospital, Quanzhou, Fujian, China; ^2^Department of Neurology, First Hospital of Quanzhou Affiliated to Fujian Medical University, Quanzhou, China; ^3^Fujian Provincial Key Laboratory of Brain Aging and Neurodegenerative Diseases, Laboratory of Clinical Applied Anatomy, School of Basic Medical Sciences, Fujian Medical University, Fuzhou, Fujian, China; ^4^Department of Infectious Disease, Quanzhou First Hospital, Quanzhou, Fujian, China

**Keywords:** nurses, burnout, sleep problems, cumulative effect, unique effects

## Abstract

**Objective:**

To investigate the unique and cumulative associations of different sleep problem subtypes with burnout among Chinese nurses.

**Methods:**

A survey was conducted in Quanzhou, China, and a total of 744 nurses were included. Burnout was measured by Maslach Burnout Inventory-Human Services Survey across three dimensions: emotional exhaustion (EE), depersonalization (DP) and low personal accomplishment (PA). Pittsburgh Sleep Quality Index was used to measure 15 types of sleep problems. Binary logistic regression was employed to explore the association between sleep problems and burnout.

**Results:**

Prevalence of sleep problems, high EE, high DP and low PA were 43.3, 6.9, 23.4, and 63.2%, respectively. Experience of sleep problems significantly increased risk for EE (*OR* = 1.31, 95%*CI*: 1.185–1.436), DP (*OR* = 1.08, 95%*CI*: 1.023–1.142) and PA (*OR* = 1.09, 95%*CI*: 1.036–1.146). Of the 15 sleep problem subtypes, “feel too cold” and “have pain” were significant predictors of high EE (*OR* = 3.89, 95%*CI*: 1.629–9.302; *OR* = 3.00, 95%*CI*: 1.314–6.827, respectively), and “daytime dysfunction” significantly predicted low PA (*OR* = 1.68, 95%*CI*: 1.135–2.491). Around 40.9% of nurses had reported more than three subtypes of sleep problems. Experiencing more than three subtypes of sleep problems were significantly associated with an increased risk of DP and PA (ORs range from 2.07 to 2.71).

**Conclusion:**

These findings suggested that sleep problems was associated with an increased risk of burnout in nurses, with both unique and cumulative risks. Interventions should focus on the prevention and management of the effects of sleep problems among nurses.

## 1 Introduction

Burnout is defined by the Maslach Burnout Inventory (MBI) as encompassing three primary dimensions: a loss of enthusiasm for work (emotional exhaustion, EE), negative feelings and cynical attitudes (depersonalization, DP), and low sense of personal accomplishment (low personal accomplishment, PA) (WHO, 2024; Maslach and Jackson, 1981; Maslach et al., 2001; Maslach and Leiter, 2016). This encompasses not only perception but also a decline in performance. The prevalence of burnout among nurses has reached epidemic levels, prompting a widespread focus on this issue (Owuor et al., 2020; Woo et al., 2020). In comparison to other healthcare professionals, nurses are more susceptible to burnout, due to their proximity to patients and the frequency of their interpersonal interactions (Westergren and Lindberg, 2023). A number of studies have indicated that burnout is a prevalent issue among nurses (Owuor et al., 2020; Woo et al., 2020). A meta-analytic study revealed that 31% of nurses exhibited high EE, while 24 and 38% of subjects demonstrated high DP and low PA, respectively (Molina-Praena et al., 2018). A study conducted in Shandong, China, revealed that 63.8% of nurses employed in general hospitals exhibited at least one symptom of burnout. Furthermore, 34.4% of the nurses in the study met the criteria for high EE, 42.2% for high DP, and 26.1% for low PA (Sun et al., 2018).

The adverse effects of nurse burnout have been documented in the literature, including an impact on nurses’ well-being, a negative effect on nurse turnover rates, and a potential threat to patient safety (Sullivan et al., 2022). Furthermore, burnout can result in suboptimal job performance (Adriaenssens et al., 2017) and a degradation in the quality of care or service provided by the nursing staff (Cañadas-De la Fuente et al., 2015). The ramifications of burnout are detrimental to nurses, patients, and healthcare institutions. Consequently, it is imperative to identify risk factors for burnout in nurses and to implement targeted interventions to prevent its occurrence.

Previous research has identified the primary risk factors for nurse burnout as being associated with stressful work environments and heavy workloads, including prolonged work hours (Woo et al., 2020; Chen and Meier, 2021). More recent studies have focused on the issue of sleep problems. Empirical evidence from both the general population and healthcare professionals indicates that the presence of sleep problems, such as insufficient sleep and poor sleep quality, may increase the odds of burnout (Wang et al., 2020; Saintila et al., 2024). Given the nature of the nurses working conditions, which often include shift work and extended hours, sleep problems are highly prevalent among nurses. This is accompanied by alterations in sleep architecture and a reduction in the quality and quantity of sleep hours (Yazdi et al., 2014; Geiger-Brown et al., 2012; Miguez-Torres et al., 2021; Wang et al., 2020). A meta-analysis comprising 12 studies has revealed a significant positive correlation between sleep problems and burnout among nurses (Membrive-Jiménez et al., 2022). In addition, both sleep duration and sleep quality have been linked to an elevated risk of burnout in surveys of nurses (Membrive-Jiménez et al., 2022; Chin et al., 2015). Although these studies provide preliminary insights into the potential relationship between sleep problems and burnout among nurses, they have also identified several key research gaps.

Firstly, sleep problems can be characterized by multiple subtypes, including poor sleep quality, high sleep latency, short sleep duration, impaired sleep efficiency, daytime dysfunction, the use of sleep medication and nine types of sleep disturbances (Cho et al., 2015; Becker et al., 2018). Nevertheless, few studies have examined the differences in the associations between various subtypes of sleep problems and burnout. This prevents psychologists from acknowledging whether and to what extent specific sleep problems subtypes are greater risk factors for burnout than others. Therefore, further research is required to examine the unique effects of all potential sleep problems subtypes on burnout among nurses, with a view to obtaining a more accurate understanding of the associations involved.

Secondly, it is not uncommon for individuals to present with multiple subtypes of sleep problems concurrently (Blunden et al., 2004; Owens et al., 2014). Additionally, a significant number of participants have indicated that they have experienced more than one subtype of sleep problem (Roth et al., 2006). Moreover, a study indicated that nurses may be susceptible to the cumulative effects of various types of sleep problems (Ma et al., 2024). It is therefore unclear whether individuals presenting with multiple subtypes of sleep problems are at an elevated risk of burnout relative to those exhibiting a single sleep problem. To date, no study has investigated whether nurses presenting with multiple types of sleep problems are more likely to engage in burnout than those presenting with a single type of sleep problem. Furthermore, there has been no prior study that has investigated the cumulative effect of multiple subtypes of sleep problems on burnout among clinical nurses, making such an investigation a clear research priority.

The objective of this study is to examine the associations between sleep problems and burnout in a large and representative sample of nurses from Fujian Province, China. Additionally, the study aims to investigate the unique and cumulative effects of different subtypes of sleep problems on three dimensions of burnout among Chinese nurses and identify the influence of depressive symptoms on this relationship (Chen and Meier, 2021).

## 2 Materials and methods

### 2.1 Study design and participants

The present cross-sectional survey was conducted from May to June 2020 in Quanzhou City, Fujian Province, China. A convenience sampling method was employed for individuals working closely with the authors or researchers involved in this study. The link to the questionnaire and invitation letters, which provided a brief introduction to the purpose and procedure of this survey, were sent to the group leaders of 50 WeChat groups. The group leaders were requested to disseminate this information to their respective WeChat groups, which ranged in size from 18 to 46 nurses. All research instruments were uploaded to the online platform Wenjuanxing^1^, which is designed for the administration of electronic questionnaires. The following individuals were eligible to participate in the study: (1) Those in possession of vocational qualification certificates and employed in a hospital setting; (2) free of dyslexia. The following nurses were excluded from the study: (1) Those with a history of mental health problems; (2) Those currently receiving psychotherapy or taking antipsychotic medication; (3) Nursing students and visiting scholars.

The minimum sample size was calculated using the single population proportion formula: $n={Z_{\alpha /2}}^{2}p\left(1-p\right)/d^{2}$. Based on the assumption that the prevalence of high EE was 31%, high DP was 24%, and low PA was 38% among nurses (Molina-Praena et al., 2018), with a marginal error of 5, a 95% confidence interval (CI) (*α* = 0.05), and a non-response rate of 20%, the final sample size was 336 nurses [280 × (1 + 0.2)].

### 2.2 Measures

#### 2.2.1 Sleep problems

The Pittsburgh Sleep Quality Index (PSQI) (Buysse et al., 1989) was used to assess sleep problems over the past month. The PSQI has been translated into Chinese, and has been widely used in Chinese nurses samples, exhibiting excellent reliability and validity (Sun et al., 1996). The PSQI comprises 18 items and seven components: subjective sleep quality, sleep latency, sleep duration, habitual sleep efficiency, sleep disturbances, use of sleep medication and daytime dysfunction. The “sleep disturbances” component of the PSQI is based on nine different types of sleep disturbances, including night/early morning waking, using bathroom, cannot breathe comfortably, cough or snore loudly, feeling too cold, feeling too hot, bad dreams, pain and other sleep disturbances. Each subtype is scored from “0” (*never*) to “3” (*at least three times a week*). The total score ranged from 0 to 21 points, with a score higher than 7 serving as the cut-off point for the presence of a sleep problem. The scores for each subtype of sleep problem (including nine different types of sleep disturbance, as well as six additional domains of the PSQI) were dichotomised into two categories: no (score of 0–1) and yes (score of 2–3). Participants with a score of 2 or above were considered to have a specific sleep problem in that domain, in accordance with the criteria established by reference to the original study (Yu et al., 2017). In this study, the Chinese version of the PSQI exhibited good internal consistency (Cronbach’s *α* = 0.88). The prevalence and severity of sleep problems were examined across three levels: with/without sleep problems, sleep problem severity represented by the global PSQI score, and 15 different types of sleep problems (nine sleep disturbances and other six domains of PSQI).

#### 2.2.2 Burnout

The Maslach Burnout Inventory-Human Services Survey (MBI-HSS) (Leiter and Harvie, 1996) is a self-administered questionnaire comprising of 22 items that assess three conceptual dimensions: emotional exhaustion (EE: 9 items), depersonalization (DP: 5 items), and low sense of personal accomplishment (PA: 8 items). The answers are given on a 7-point Likert scale (0 = *never*, 6 = *every day*). The scale does not generate an overall score, but the scores can be dichotomized into high EE (≥27 points), high DP (≥8 points) and low PA (≤24 points) (Ye et al., 2008). The presence of high levels of EE and DP and low PA is indicative of the burnout syndrome. In this study, the MBI-HSS exhibited good internal consistency, with Cronbach’s *α* coefficient was 0.925, 0.850 and 0.901 for EE, DP and PA, respectively.

#### 2.2.3 Depressive symptoms

In addition, depressive symptoms that have been demonstrated to potentially influence the development of burnout were measured and controlled for (Chen and Meier, 2021). The Patient Health Questionnaire-9 (PHQ-9) is a nine-item scale to assess the severity of depressive symptoms within the past 2 weeks. Each item is rated on a 4-point Likert scale, ranging from “0” (*not at all*) to “3” (*nearly every day*). The sum scores ranged from 0 to 27, with a cut-off score of 5 or above indicating the presence of depressive symptoms (Kroenke et al., 2001). In the present study, the Cronbach’s alpha coefficient was 0.922.

#### 2.2.4 Sociodemographic and occupational characteristics

The present study considered a number of sociodemographic items, including age (25 years old or below, 26–35, 35–45, or above 46 years old), gender (males, females), marital status (married or living with a partner, or other), parental status (yes, no), location (rural, suburban, urban), ethnicity (minority nationality, the Han nationality), education background (junior college or less, bachelor’s degree or above), body mass index (BMI, <18.5, 18.5 ~ 23.9, ≥24), monthly income (4,000 yuan or below, above 4,000 yuan). The occupational characteristics of the participants were assessed, including their employment status (formal employee, contract employee), professional status (registered nurse, primary, intermediate, senior or deputy senior), position title (nurse, head nurse), and years of nursing practice (5 years or less, 6–10 years, 11 years or more). The body mass index (BMI) was computed with the following formula: BMI = kg/m^2^.

### 2.3 Statistical analysis

All analyses were conducted with SPSS statistical software version 25.0. Continuous variables are reported as means ± standard deviation (M ± SD), and categorical variables are summarized as frequencies (*n*) and percentages (%). Spearman’s correlation coefficient correlation was employed to analyze the correlation between the various subtypes of sleep problems. Univariate logistic regression analyses were conducted to examine the relationship between the sociodemographic and occupational characteristics of nurses and each of the three dimensions of burnout separately. Hierarchical logistic multiple regression models were subsequently utilized to investigate the relationships between sleep problems and each of the three dimensions of burnout. Regression models included the following independent variables separately: without/with sleep problems (Model 1), the PSQI global score (Model 2), and 15 different subtypes of sleep problems (Model 3). Apart from the raw model, regression models were further conducted with adjustments for covariates that showed statistically significant associations with the three dependent variables in the univariate logistic regression analyses, and depressive symptoms. To further explore the cumulative effects of different subtypes of sleep problems on three dimensions of burnout, we categorized nurses into five groups as follows: (1) no sleep problem (reference), (2) one sleep problem, (3) two sleep problems, (4) three sleep problems, and (5) four or more sleep problems.

The results were expressed as odds ratios (OR) and 95% confidence intervals (95%CI) for all logistic regression models. Statistical significance was assessed at the 2-tailed 5% level.

### 2.4 Ethical considerations

This study is in accordance with the ethical conduct of human subjects research in Declaration of Helsinki and was approved by the Ethics Committee of Quanzhou First Hospital, Fujian Province China (No. Quan Yi lun2020253). Prior to their participation, all participants provided online informed consent.

## 3 Results

### 3.1 Sociodemographic and occupational characteristics and associations with burnout

The survey comprised 744 nurses with the average age of 31.13 ± 6.39 years old. The majority of the sample (97%, 722/744) were female, and 350 (47.0%) of the participants reported depressive symptoms. Around 6.9% (*n* = 51) of sample reported high EE, 23.4% (*n* = 174) reported high rates of DP, and 63.2% (*n* = 470) reported low PA. Table 1 shows that experiencing depression statistically elevated risk of high EE, high DP, and low PA in nurses. At *p* < 0.05 significant level, monthly income over 4,000 yuan reduced the risk of high EE. Primary professional status was associated with high DP. Contract employee employment status was associated with low PA. And the lower risk of low PA was statistically associated with the following characteristics: over 45 years of age, married or living with a partner, having children, having intermediate professional status having head nurse position title, and more than 11 years of nursing practice. More details are provided in Table 1.

**Table 1:** Population characteristics for nurses in this study and their associations with Burnout (*N* = 744).

Characteristics	Total sample (*N* = 744)	Emotional exhaustion (EE)	Depersonalization (DP)	Low personal accomplishment (PA)
	*n* (%)	OR(95%CI)	OR(95%CI)	OR(95%CI)
Age (years old, Mean ± SD)	31.13 ± 6.39			
≤25	139(18.7)	1	1	1
26–35	457(61.4)	1.23(0.55–2.74)	1.43(0.89–2.28)	1.08(0.73–1.61)
36–45	116(15.6)	1.21(0.44–3.34)	0.97(0.52–1.82)	0.77(0.46–1.27)
≥46	32(4.3)	1.69(0.42–6.78)	1.38(0.56–3.41)	0.38(0.18–0.84) ^*^
Gender				
Male	22(3.0)	1	1	1
Female	722(97.0)	NA	0.65(0.26–1.61)	1.75(0.75–4.08)
Marital status				
Other	253(34.0)	1	1	1
Married/Live with a partner	491(66.0)	1.26(0.67–2.34)	1.37(0.95–1.99)	0.69(0.50–0.95) ^*^
Parental status				
No	286(38.4)	1	1	1
Yes	458(61.6)	1.16(0.64–2.10)	1.06(0.75–1.51)	0.67(0.49–0.91) ^*^
Location				
Rural	131(17.6)	1	1	1
Suburban	122(16.4)	0.84(0.40–1.76)	0.94(0.60–1.49)	0.79(0.52–1.18)
Urban	491(66.0)	0.96(0.38–2.46)	1.15(0.65–2.03)	0.90(0.53–1.51)
Ethnicity				
Minority	8(1.1)	1	1	1
Han	736(98.9)	0.51(0.06–4.23)	0.92(0.18–4.57)	2.89(0.69–12.20)
Education background				
Junior college or below	396(53.2)	1	1	1
Bachelor degree or above	348(46.8)	0.78(0.44–1.40)	1.26(0.90–1.77)	0.89(0.66–1.21)
BMI				
<18.5	119(16.0)	1	1	1
18.5 ~ 23.9	497(66.8)	1.73(0.66–4.51)	0.74(0.47–1.17)	0.82(0.54–1.26)
≥24	128(17.2)	2.14(0.72–6.36)	0.83(0.47–1.47)	0.76(0.45–1.28)
Monthly income				
≤4000yuan	237(31.9)	1	1	1
>4,000 yuan	507(68.1)	0.54(0.31–0.97)	1.02(0.71–1.46)	0.92(0.66–1.26)
Employment status				
Formal employee	341(45.8)	1	1	
Contract employee	399(53.6)	0.81(0.46–1.43)	0.72(0.51–1.02)	1.38(1.02–1.86) ^*^
Missing	4(0.5)	NA	NA	NA
Professional status				
Registered nurse	226(30.4)	1	1	
Primary	328(44.1)	1.75(0.85–3.61)	1.56(1.03–2.36) ^*^	0.89(0.62–1.27)
Intermediate	175(23.5)	1.44(0.62–3.34)	1.18(0.72–1.93)	0.59(0.39–0.89) ^*^
Senior/deputy senior	10(1.3)	NA	2.84(0.77–10.49)	0.49(0.14–1.73)
Missing	5(0.7)	NA	NA	NA
Position title				
Nurse	695(93.4)	1	1	1
Head nurse	40(5.4)	1.57(0.54–4.60)	0.94(0.44–2.02)	0.52(0.27–0.98) ^*^
Missing	9(1.2)	NA	NA	NA
Years of nursing practice				
≤5	246(33.1)	1	1	1
6 ~ 10	311(41.8)	1.64(0.82–3.25)	1.18(0.79–1.74)	0.95(0.66–1.35)
≥11	187(25.1)	1.23(0.55–2.76)	0.87(0.54–1.38)	0.53(0.36–0.79) ^**^
Depression status				
No depression	394(53.0)	1	1	1
Depression	350(47.0)	65.50(9.00–476.86) ^***^	5.72(3.85–8.48) ^***^	2.18(1.60–2.97) ^***^

*p < 0.05, **p < 0.01, ***p < 0.001; NA, not applicable or data available.

### 3.2 Correlations among different sleep problem subtypes

As shown in Table 2, 322(43.3%) of all nurses reported having experienced sleep problems with a PSQI global score over 7. Among the different subtypes of sleep problems assessed using PSQI, the most common was “short sleep duration” (49.1%), followed by “daytime dysfunction” (45.7%), “difficulty falling sleep” (42.3%), and “poor sleep quality” (39.7%).

**Table 2:** Correlation of overall sleep problems with different subtypes of sleep problems and their prevalence.

Specific subtypes of sleep problems	1	2	3	4	5	6	7	8	9	10	11	12	13	14	15	16
1. Overall sleep problems	1	0.701^***^	0.613^***^	0.494^***^	0.347^***^	0.509^***^	0.332^***^	0.215^***^	0.246^***^	0.237^***^	0.317^***^	0.417^***^	0.237^***^	0.445^***^	0.209^***^	0.582^***^
2. Poor sleep quality		1	0.507^***^	0.304^***^	0.198^***^	0.495^***^	0.331^***^	0.248^***^	0.153^***^	0.162^***^	0.246^***^	0.371^***^	0.213^***^	0.384^***^	0.163^***^	0.492^***^
3. Difficulty falling sleep			1	0.275^***^	0.160^***^	0.447^***^	0.318^***^	0.175^***^	0.161^***^	0.161^***^	0.240^***^	0.306^***^	0.165^***^	0.347^***^	0.136^***^	0.339^***^
4. Short sleep duration				1	0.305^***^	0.247^***^	0.110^**^	0.150^***^	0.106^**^	0.088^*^	0.119^**^	0.163^***^	0.064	0.215^**^	0.080^*^	0.282^***^
5. Impairment of sleep efficiency					1	0.108^**^	0.137^***^	0.040	0.066	0.036	0.101^**^	0.068	0.024	0.073^*^	0.037	0.109^**^
6. Night/early morning waking						1	0.485^***^	0.222^***^	0.227^***^	0.227^***^	0.295^***^	0.406^***^	0.202^***^	0.454^***^	0.122^***^	0.440^***^
7. Have to use bathroom							1	0.231^***^	0.239^***^	0.277^***^	0.290^***^	0.274^***^	0.156^***^	0.262^***^	0.112^**^	0.284^***^
8. Cannot breathe comfortably								1	0.256^***^	0.199^***^	0.284^***^	0.228^***^	0.205^***^	0.223^***^	0.111^**^	0.201^***^
9. Cough or snore loudly									1	0.323^***^	0.274^***^	0.249^***^	0.296^***^	0.226^***^	0.017	0.229^***^
10. Feel too cold										1	0.347^***^	0.403^***^	0.313^***^	0.306^***^	−0.008	0.266^***^
11. Feel too hot											1	0.317^***^	0.277^***^	0.286^***^	0.038	0.287^***^
12. Have bad dreams												1	0.314^***^	0.461^***^	0.112^**^	0.385^***^
13. Have pain													1	0.363^***^	0.028	0.212^***^
14. Other sleep disturbances														1	0.024	0.426^***^
15. Use of sleeping medication															1	0.153^***^
16. Daytime dysfunction																1
*n*(%)	322(43.3)	295(39.7)	315(42.3)	365(49.1)	78(10.5)	286(38.4)	250(33.6)	46(6.2)	72(9.7)	72(9.7)	127(17.1)	134(18.0)	62(8.3)	147(19.8)	24(3.2)	340(45.7)

*p < 0.05, **p < 0.01, ***p < 0.001.

### 3.3 Association between overall sleep problems and burnout

As presented in Models 1 shown in Tables 3–5, nurses who experienced overall sleep problems (global PSQI score over 7) had a significantly higher risk of high EE, high DP, and low PA, after adjusting for significant sociodemographic and occupational characteristics. When depression was controlled for, the associations were attenuated but remained statistically significant.

**Table 3:** Associations between sleep problems and Emotional Exhaustion.

		Unadjusted OR (95%CI)	*p-*value	Adjusted OR ^a^(95%CI)	*P-*value	Adjusted OR ^b^(95%CI)	*P-*value
Model 1	PSQI global score (categorical)						
	Without sleep problems	1[Reference]		1[Reference]		1[Reference]	
	With sleep problems	7.976 (3.694–17.222)	**<0.001**	8.248(3.809–17.861)	**<0.001**	3.631(1.631–8.084)	**0.002**
Model 2	PSQI global score (continuous)	1.409(1.291–1.537)	**<0.001**	1.417(1.298–1.548)	**<0.001**	1.305(1.185–1.436)	**<0.001**
Model 3	Specific subtypes of sleep problems						
	Poor sleep quality	2.558(0.988–6.624)	0.053	2.461(0.938–6.458)	0.067	2.267(0.856–6.007)	0.100
	Difficulty falling sleep	1.061(0.466–2.412)	0.888	1.105(0.481–2.539)	0.813	0.987(0.419–2.325)	0.976
	Short sleep duration	1.987(0.877–4.502)	0.100	1.996(0.875–4.551)	0.100	1.942(0.831–4.537)	0.126
	Impairment of sleep efficiency	0.799(0.297–2.152)	0.657	0.764(0.284–2.055)	0.594	0.716(0.264–1.943)	0.512
	Night/early morning waking	1.605(0.604–4.266)	0.343	1.704(0.633–4.589)	0.292	1.734(0.628–4.787)	0.288
	Have to use bathroom	0.605(0.272–1.345)	0.218	0.577(0.257–1.297)	0.183	0.56(0.24–1.306)	0.179
	Cannot breathe comfortably	2.311(0.917–5.824)	0.076	2.373(0.93–6.057)	0.071	1.939(0.755–4.975)	0.169
	Cough or snore loudly	1.877(0.802–4.391)	0.147	1.972(0.835–4.657)	0.121	1.749(0.709–4.317)	0.225
	Feel too cold	3.893(1.629–9.302)	**0.002**	4.127(1.711–9.953)	**0.002**	4.24(1.743–10.311)	**0.001**
	Feel too hot	1.486(0.679–3.25)	0.322	1.502(0.679–3.321)	0.316	1.39(0.63–3.066)	0.415
	Have bad dreams	0.765(0.333–1.756)	0.527	0.68(0.289–1.595)	0.375	0.597(0.254–1.402)	0.236
	Have pain	2.995(1.314–6.827)	**0.009**	2.674(1.156–6.187)	**0.022**	2.83(1.182–6.775)	**0.019**
	Other sleep disturbances	1.849(0.824–4.151)	0.136	1.954(0.862–4.431)	0.109	1.754(0.76–4.048)	0.188
	Use of sleeping medication	3.169(0.898–11.183)	0.073	3.613(1.024–12.748)	**0.046**	3.661(0.983–13.636)	0.053
	Daytime dysfunction	1.415(0.53–3.777)	0.488	1.546(0.567–4.217)	0.395	1.041(0.365–2.967)	0.940

OR, odds ratio; CI, confidence interval. ^a^Adjusted for monthly income. ^b^Further adjusted for depression status. Bold indicates statistical significance.

**Table 4:** Associations between sleep problems and Depersonalization.

		Unadjusted OR (95%CI)	*P-*value	Adjusted OR ^a^(95%CI)	*P-*value	Adjusted OR ^b^(95%CI)	*P-*value
Model 1	PSQI global score (categorical)						
	Without sleep problems	1[Reference]		1[Reference]		1[Reference]	
	With sleep problems	3.200(2.244–4.563)	**<0.001**	3.152(2.207–4.502)	**<0.001**	1.786(1.204–2.65)	**0.004**
Model 2	PSQI global score (continuous)	1.181(1.126–1.239)	**<0.001**	1.179(1.124–1.237)	**<0.001**	1.081(1.023–1.142)	**0.005**
Model 3	Specific subtypes of sleep problems						
	Poor sleep quality	1.558(0.976–2.489)	0.063	1.608(1.002–2.581)	**0.049**	1.346(0.828–2.188)	0.231
	Difficulty falling sleep	1.204(0.785–1.847)	0.395	1.201(0.779–1.85)	0.407	1.131(0.725–1.762)	0.588
	Short sleep duration	1.018(0.683–1.516)	0.932	1.01(0.675–1.51)	0.963	0.911(0.6–1.383)	0.662
	Impairment of sleep efficiency	1.378(0.786–2.413)	0.263	1.363(0.774–2.399)	0.283	1.253(0.705–2.226)	0.443
	Night/early morning waking	1.058(0.656–1.708)	0.817	1.045(0.642–1.701)	0.858	0.936(0.567–1.544)	0.794
	Have to use bathroom	0.925(0.599–1.428)	0.725	0.904(0.585–1.399)	0.651	0.95(0.605–1.489)	0.822
	Cannot breathe comfortably	1.319(0.659–2.644)	0.434	1.322(0.656–2.662)	0.435	1.111(0.551–2.242)	0.769
	Cough or snore loudly	1.036(0.566–1.898)	0.908	1.066(0.581–1.958)	0.836	1.06(0.568–1.98)	0.855
	Feel too cold	1.399(0.753–2.601)	0.289	1.381(0.741–2.574)	0.309	1.21(0.644–2.274)	0.553
	Feel too hot	1.324(0.813–2.155)	0.260	1.307(0.801–2.135)	0.284	1.202(0.728–1.986)	0.472
	Have bad dreams	1.583(0.952–2.632)	0.077	1.613(0.963–2.701)	0.069	1.452(0.858–2.456)	0.165
	Have pain	1.039(0.543–1.99)	0.908	1.017(0.529–1.957)	0.960	1.019(0.523–1.984)	0.956
	Other sleep disturbances	0.737(0.438–1.24)	0.251	0.711(0.421–1.202)	0.203	0.683(0.4–1.165)	0.162
	Use of sleeping medication	1.13(0.462–2.769)	0.788	1.033(0.418–2.549)	0.944	1.045(0.415–2.631)	0.926
	Daytime dysfunction	1.582(1.017–2.461)	**0.042**	1.635(1.045–2.556)	**0.031**	1.226(0.765–1.964)	0.398

^a^Adjusted for Professional status. bFurther adjusted for depression status. Bold indicates statistical significance.

**Table 5:** Associations between sleep problems and low sense of Personal Accomplishment.

		Unadjusted OR (95%CI)	*P-*value	Adjusted OR ^a^(95%CI)	*P-*value	Adjusted OR ^b^(95%CI)	*P-*value
Model 1	PSQI global score (categorical)						
	Without sleep problems	1[Reference]		1[Reference]		1[Reference]	
	With sleep problems	2.381(1.738–3.262)	**<0.001**	2.483(1.794–3.435)	**<0.001**	2.018(1.414–2.88)	**<0.001**
Model 2	PSQI global score (continuous)	1.120(1.072–1.169)	**<0.001**	1.125(1.076–1.176)	**<0.001**	1.09(1.036–1.146)	**0.001**
Model 3	Specific subtypes of sleep problems						
	Poor sleep quality	1.466(0.955–2.25)	0.080	1.437(0.925–2.233)	0.107	1.331(0.85–2.083)	0.211
	Difficulty falling sleep	1.599(1.085–2.358)	**0.018**	1.49(0.998–2.224)	0.051	1.461(0.975–2.19)	0.066
	Short sleep duration	0.979(0.696–1.376)	0.903	1.05(0.738–1.495)	0.785	1.01(0.706–1.443)	0.958
	Impairment of sleep efficiency	1.972(1.073–3.621)	**0.029**	1.916(1.031–3.562)	**0.040**	1.818(0.976–3.387)	0.059
	Night/early morning waking	0.917(0.591–1.422)	0.699	0.993(0.630–1.565)	0.975	0.954(0.602–1.512)	0.841
	Have to use bathroom	0.897(0.603–1.333)	0.590	0.854(0.568–1.282)	0.445	0.867(0.576–1.307)	0.496
	Cannot breathe comfortably	1.521(0.7–3.303)	0.289	1.798(0.810–3.993)	0.150	1.65(0.741–3.674)	0.220
	Cough or snore loudly	0.860(0.473–1.566)	0.623	0.999(0.536–1.863)	0.997	0.989(0.53–1.845)	0.971
	Feel too cold	1.169(0.615–2.22)	0.634	1.202(0.624–2.314)	0.582	1.129(0.584–2.183)	0.719
	Feel too hot	1.258(0.768–2.061)	0.362	1.203(0.724–2.00)	0.475	1.161(0.696–1.934)	0.568
	Have bad dreams	0.918(0.545–1.545)	0.747	0.859(0.504–1.464)	0.576	0.812(0.474–1.393)	0.449
	Have pain	0.866(0.457–1.641)	0.660	0.841(0.439–1.613)	0.602	0.829(0.433–1.589)	0.573
	Other sleep disturbances	0.514(0.309–0.855)	**0.010**	0.514(0.305–0.866)	**0.012**	0.5(0.296–0.845)	**0.010**
	Use of sleeping medication	0.669(0.267–1.674)	0.390	0.737(0.284–1.912)	0.530	0.734(0.281–1.918)	0.528
	Daytime dysfunction	1.681(1.135–2.491)	**0.010**	1.758(1.169–2.642)	**0.007**	1.6(1.056–2.424)	**0.027**

^a^Adjusted for age, marital status, parental status, employment status, professional status, position title, years of nursing practice. bFurther adjusted for depression status. Bold indicates statistical significance.

When the model used the PSQI global score as a continuous variable, we found that each unit increase in global PSQI score were significantly related to 31% increased risk of high EE (*OR* = 1.31, 95%*CI*:1.185–1.436), and 8% increased risk of high DP (*OR* = 1.08, 95%*CI*: 1.023–1.142), and 9% increased risk of low PA (*OR* = 1.09, 95%*CI*: 1.036–1.146), separately, after controlling for all significant sociodemographic and occupational variables, including depression (Models 2 in Tables 3–5).

### 3.4 Associations between the different sleep problem subtypes and burnout

As seen Model 3 in Table 3, we observed that “feel too cold” and “have pain” in “sleep disturbances” domains were significantly risk factors of high EE (*OR* = 3.89, 95%*CI*: 1.629–9.302; *OR* = 3.00, 95%*CI*: 1.314–6.827). When controlling for significant sociodemographic and occupational variables, the associations remained significant. When further controlling for depression, nurses who felt too cold and had pain remained significantly associated with increased risk for high EE.

When all individual subtypes of sleep problems including into the multivariate logistic model simultaneously, no association was found between any of them and high DP (Model 3 in Table 4).

As shown in Model 3 in Table 5, “daytime dysfunction” was significantly associated with increased risk of low PA (*OR* = 1.68, 95%*CI*: 1.135–2.491). When controlling for significant variables, including depression, the associations remained significant.

### 3.5 Association of cumulative sleep problems and burnout

Table 6 shows that approximately 74.1% of nurses had experienced at least one subtype of sleep problems. More specifically, 18.5% had reported experiencing one subtype of sleep problems, 14.7% had reported two subtypes of sleep problems, 40.9% of nurses had reported three or more subtypes of sleep problems.

**Table 6:** Cumulative effects of different subtypes of sleep problems on Burnout.

Total number of sleep problems	Overall	Emotional exhaustion^a^	Depersonalization			Low personal accomplishment		
				Multivariate regression			Multivariate regression	
	*n*(%)	*n*(%)	*n*(%)	Adjusted OR^b^ (95%CI)	Adjusted OR^c^ (95%CI)	*n*(%)	Adjusted OR^d^ (95%CI)	Adjusted OR^e^ (95%CI)
0 sleep problems	193(25.9)	0(0.0)	21(10.9)	1[Reference]	1[Reference]	100(51.8)	1[Reference]	1[Reference]
1 sleep problems	138(18.5)	6(4.3)	20(14.5)	1.409(0.73–2.72)	1.138(0.573–2.258)	81(58.7)	1.297(0.824–2.042)	1.225(0.774–1.937)
2 sleep problems	109(14.7)	2(1.8)	25(22.9)	2.446(1.291–4.633) **	1.771(0.904–3.469)	61(56.0)	1.174(0.724–1.905)	1.06(0.648–1.736)
3 sleep problems	112(15.1)	7(6.3)	36(32.1)	3.978(2.171–7.289) ***	2.123(1.105–4.078) *	87(77.7)	3.324(1.937–5.704) ***	2.705(1.539–4.754) **
4 or more sleep problems	192(25.8)	36(18.8)	72(37.5)	4.883(2.839–8.398) ***	2.068(1.13–3.786) *	141(73.4)	2.686(1.727–4.178) ***	1.997(1.212–3.292) **

^a^All those with EE experienced sleep problems, so analysis was not performed. ^b^Adjusted for professional status. ^c^Further adjusted for depression status; ^d^Adjusted for age, marital status, parental status, employment status, professional status, position title, years of nursing practice. ^e^Further adjusted for depression status. *p < 0.05, **p < 0.01, ***p < 0.001.

All those with high EE experienced sleep problems, so analysis was not performed. After adjusting for all potential confounders, nurses with three subtypes of sleep problems, and four or more subtypes of sleep problems were significantly associated with increased odds of high DP when compared to those without any sleep problems (*OR* = 2.12, 95%*CI*: 1.105–4.078; *OR* = 2.07, 95%*CI*: 1.13–3.79). Nurses who suffered from three and four or more subtypes of sleep problems all had increased odds of reporting low PA compared with those who did not suffer from any sleep problems. After adjusting for covariates, we observed similar results, although the risk was slightly reduced (*OR* = 2.71, 95%*CI*: 1.54–4.75; *OR* = 2.00, 95%*CI*: 1.212–3.292).

## 4 Discussion

To our knowledge, this is the first study to examine the relationship between sleep problems and three dimensions of burnout in a sample of 744 nurses from China, exploring global issues down to specific problems, and considering both unique and cumulative effects. After controlling for sociodemographic and occupational characteristics, in addition to depression, this study demonstrated that nurses experiencing sleep problems were more likely to develop high EE, high DP and low PA. Additionally, “feel too cold” and “have pain” were found to be risk factors for high EE. Moreover, “daytime dysfunction” was identified as a risk factor for low PA. Further, we found that nurses with three or more sleep problems were twice as likely to develop high DP and low PA than those with no sleep problems.

In the current study, the overall prevalence of burnout was 67.7%, which is higher than that found in a study of Chinese nurses (Sun et al., 2018). In addition, this result is much higher than the overall prevalence of burnout symptoms among nurses of 11.23% reported in a meta-analysis of 61 studies (Woo et al., 2020). Regarding the three dimensions of burnout, the prevalence of high EE (6.9%) and high DP (23.4%) were within the respective ranges of 5.6–69.6% and 1.1–39.1%, respectively, reported in a previous meta-analysis (Galanis et al., 2021). However, the prevalence of low PA (63.2%) was significantly higher than the range of 0–52.2%. This may be related to the investigation of different healthcare systems, working conditions and epidemiological severity, as well as the utilization of different assessment methods and cut-offs for burnout. Furthermore, we had to take into account the impact of the COVID-19 pandemic; increased workload, fear of infection, segregation of work and lack of understanding of some patients may have contributed to high EE, high DP, and low PA (Bruyneel et al., 2021).

Consistent with previous studies (Wang et al., 2021), the results of this study indicated that the risk of all three dimensions of burnout was higher in nurses with sleep problems than in those without. This remained the case even after taking into account participant characteristics, including depression. This may highlight the potential importance, even in the presence of other well-recognized risk factors, of assessing nurses for sleep problems in burnout prevention and intervention.

In terms of sleep problem subtypes, we found that “feel too cold” and “have pain” were significant risk factors for high EE after adjusting for a comprehensive number of confounders. Zborowska et al. (2021) demonstrated that EE is significantly influenced by disturbed sleep and environmental factors. The identification of factors such as feeling too cold and having pain in this study is consistent with the notion that environmental and physical discomfort can affect EE. This may be because feeling cold or aching while sleeping is related to sleep deprivation (e.g., frequent nocturnal awakenings, difficulty falling asleep and disrupted sleep continuity) (Finan et al., 2013). In addition, sleep is associated with emotional processing and affect regulation (Yoo et al., 2007). Neuroimaging evidence show hyper-responsiveness in the sleep-deprived amygdala, indicating increased emotional intensity after sleep deprivation (Yoo et al., 2007; Trockel et al., 2020). These finding suggest that sleep deprivation caused by feeling cold or pain (Finan et al., 2013), may lead to impaired mood and affect regulation, thus contributing to emotional exhaustion (Trockel et al., 2020; Stewart and Arora, 2019). Further, it can be reasonably deduced that nurses who are suffering from high levels of EE as a direct result of discomfort such as feeling the cold or experiencing pain may find themselves experiencing a reduction in their ability to concentrate and an increased tendency to make errors at work (Buysse et al., 1989). This can have a significant impact on patient safety and the efficiency of the workflow in question. Furthermore, chronic pain or discomfort has the potential to result in increased absenteeism among nursing staff, which in turn can lead to a decline in morale, cohesion and ultimately, the atmosphere in the workplace.

The present study also demonstrated that nurses with daytime dysfunction were more likely to develop low PA. This is analogous to a study of nurses in the UK (Yang et al., 2020), which showed that poor sleep quality and daytime dysfunction were associated with increased burnout, including low PA. Similarly, another study (Shi et al., 2023) identified a positive correlation between daytime dysfunction and high EE and DP and low PA in medical residents undergoing standardized residency training. It is noteworthy that Shi (Shi et al., 2023) observed that the proposed models linking sleep disorders, primarily daytime dysfunction, and burnout include two main hypotheses. The first hypothesis posits that burnout results from exhaustion of the body, mind, and spirit, which can be reversed by healthy sleep. The second hypothesis suggests that chronic stress hyperactivates the hypothalamic–pituitary–adrenal axis, thereby mediating both burnout and sleep loss (Stewart and Arora, 2019). It can thus be inferred that the experience of daytime dysfunction can result in a reduction in PA, which in turn can lead to a decline in work productivity and effectiveness in nursing tasks, ultimately impacting the quality of patient care. Moreover, nurses who are experiencing daytime dysfunction may also experience a reduction in their quality of life due to increased fatigue and a diminished capacity to engage in daily activities, which can affect their overall well-being and work-life balance. In our study, the relationship between daytime dysfunction and DP was not significant after adjusting for the influence of depression. One potential explanation for this is that the correlation between daytime dysfunction and depersonalization may be mediated by the status of depressive symptoms.

We also found that, compared with those with no sleep problems, nurses with three or more subtypes of sleep problems were associated with a twofold risk of developing high DP and low PA. This finding is consistent with the cumulative risk model, which postulates that individuals often encounter multiple independent risk factors rather than a single factor, and that the occurrence of additive risk factors intensifies the likelihood of unexpected outcomes (Sameroff, 2000). To date, our study is the first to report the cumulative effect of multiple sleep problem subtypes on nurse burnout. Notwithstanding the observation in our study that other subtypes of sleep problems are not significantly associated with any dimension of burnout, their cumulative effect on burnout remains substantial. Therefore, a comprehensive assessment of nurses’ sleep problems is needed facilitate the timely prevention and effective reduction of nurse burnout.

The findings of this study contribute to the existing evidence base for clinical practice. The findings suggest that sleep-focused burnout prevention and intervention programs may be an effective strategy for reducing burnout among nurses. Firstly, this study indicates the existence of discrete subtypes of sleep problems among nurses, highlighting the need for future research on sleep problem subtypes, targeted prevention and intervention rather than a one-size-fits-all approach to reducing burnout among nurses. In addition, it is recommended that particular attention be paid to sleep disturbances (e.g., “feeling too cold” and “having pain”) and daytime dysfunction, as they play an important role in the development of high EE or low PA in nurses. Hospital administrators can play a crucial role in mitigating the negative health effects associated with poor sleep by educating nurses about sleep hygiene. They should emphasize the importance of maintaining good sleep hygiene and provide strategies to improve sleep quality. Key recommendations include maintaining a consistent sleep schedule, creating a conducive sleep environment, and avoiding caffeine and electronic devices before bedtime. Furthermore, although there was no significant association between other subtypes of sleep problems and all three dimensions of burnout, their cumulative effect and burnout remained significant. This highlights the necessity for a comprehensive assessment of sleep problem subtypes among nurses in order to facilitate the timely prevention and effective reduction of burnout.

## 5 Limitations

A few limitations of the study should be noted when considering its contributions to existing research and the design of future studies. First, the cross-sectional nature of the study design precludes the possibility of inferring specific causal relationships. Second, although participants in this study were anonymous, identification may have biased their questionnaire responses, and our study variables were measured using self-reported data, which may introduce biases. Third, as potential factors contributing to burnout, we only assessed depression and no other factors, particularly job strain (Metlaine et al., 2017). Fourth, the recruitment of nurses from a single geographical location, namely Quanzhou City in China, may have restricted the generalizability of the findings to other urban settings. Finally, the present study was conducted with a sample of nurses in tertiary hospitals only, which may limit the generalizability of the results to other primary and secondary hospitals.

## 6 Conclusion

Despite the above limitations, this is the first study to test the relationship between sleep problems and three dimensions of burnout among Chinese nurses, exploring global issues down to specific problems, and considering both unique and cumulative effects. The findings indicated that compared with those without sleep problems, nurses with sleep problems, especially those with multiple subtypes of sleep problems, were more likely to experience burnout. In the present study, we found that feeling cold or painful during sleep predicted higher EE, and poor daytime function predicted lower PA. A previous study suggested that sleep hygiene interventions may have beneficial effects on burnout (Brubaker et al., 2020). These findings may guide future research to design prevention and intervention tailored to nurses with distinct sleep problem subtypes, which may be a way forward in combating burnout. In addition, focusing on the cumulative effects of sleep problems may provide an overall picture of the relationship between sleep problems and burnout in nurses. Future studies are needed to further confirm our findings in a longitudinal design.

## Data Availability

The raw data supporting the conclusions of this article will be made available by the authors, without undue reservation.

## Footnotes

^1^ https://www.wjx.cn
